# Embedded delta shocks

**DOI:** 10.1016/j.heliyon.2020.e04152

**Published:** 2020-06-05

**Authors:** Pablo Castañeda

**Affiliations:** Department of Mathematics, ITAM, Río Hondo 1, Ciudad de México 01080, Mexico

**Keywords:** Applied mathematics, Theoretical fluid dynamics, Mathematical analysis, Magnetohydrodynamics, Chromatography, Conservation laws, Riemann problem, Delta shock, Singular solutions, Rankine-Hugoniot, Two-phase flow

## Abstract

In 1977 Korchinski presented a new type of shock discontinuity in conservation laws. These singular solutions were coined *δ*-shocks since there is a time dependent Dirac delta involved. A naive description is that such *δ*-shock is of the overcompressive type: a single shock wave belonging to both families, the four characteristic lines of which impinge into the shock itself. In this work, we open the fan of solutions by studying two-family waves without intermediate constant states but possessing central rarefactions or comprising *δ*-shocks.

## 1 Introduction

The introduction of *δ*-shocks occurred forty years ago with the unpublished thesis [1], where such discontinuities appear in a theoretical context. Around that time, there was a simplified model for multiphase flow in porous media due to D.W. Peaceman that also presented such a mass accumulation within one of these singularities, [2]. Along these four decades, the applicability of *δ*-shocks have emerged in many areas such as chromatography [3], [4], magnetohydrodynamics [5], [6], [7], traffic flow [8], fluid dynamics [9], and perhaps also in flow in porous media [10], and other areas.

It is natural to consider a *δ*-shock with speed *σ* as an *overcompressive shock wave*, which means a discontinuity satisfying that left and right characteristic lines impinge into the shock itself, *i.e.*,

(1) $$\lambda_{1,2}(U_{L})\;>\;\sigma \;>\;\lambda_{1,2}(U_{R}),$$

for $U_{L}={\left(u_{L},\;v_{L}\right)}^{⊤}$ and $U_{R}={\left(u_{R},\;v_{R}\right)}^{⊤}$ the left and right Riemann data and $\lambda_{1,2}(U)$ the characteristic speeds for a point $U={\left(u,\;v\right)}^{⊤}$ in state space; *cf.* [1], [5], [6], [8], [11], [12], [13]. Overcompressibility in Eq. (1) is a natural extension of Lax classification, [14], which considers also the discontinuities satisfying the following speed inequalities

(2) $$\lambda_{1,2}(U_{L})\;>\;\;\sigma \;>\;\lambda_{1}(U_{R}),\;\lambda_{2}(U_{R})\;\geq \;\;\sigma ,$$

(3) $$\lambda_{2}(U_{L})\;>\;\;\sigma \;>\;\lambda_{1,2}(U_{R}),\;\;\sigma \;\geq \;\lambda_{1}(U_{L}),$$

(4) $$\lambda_{2}(U_{L})\;>\;\;\sigma \;>\;\lambda_{1}(U_{R}),\;\lambda_{2}(U_{R})\;\geq \;\;\sigma \;\geq \;\lambda_{1}(U_{L}),$$

giving rise to *1-Lax shock waves* in Eq. (2), *2-Lax shock waves* in Eq. (3), and *undercompressive* or *transitional shock waves* in Eq. (4). Left- and right-characteristic shocks are included in this shock type definition. They occur when a shock speed coincides with the characteristic speed. Whereas by definition overcompressive shocks cannot be characteristic, see Eq. (1), the limit of the inequalities above are included in (2)-(4); for further details see [15], [16], [17] and references therein.

The types of shocks given by (2)-(4) are not found explicitly in the literature in conjunction to *δ*-shocks. From the extensively large bibliographic review in [4] for models with *δ*-shocks, we notice that the conservation laws models that were identified and analyzed are weakly coupled and of the form

(5a) $$u_{t}+{\left(F(u,\;v)\right)}_{x}\;=\;0,\;\;(x,\;t)\in R\times R^{+},$$

(5b) $${\left(u^{\alpha }v\right)}_{t}+{\left(G(u,\;v)\right)}_{x}\;=\;0,\;\;(x,\;t)\in R\times R^{+},$$

where *α* is zero or one, and *F* and *G* are linear in *v*, see also [3]. We identify the Riemann solution by $U={\left(u,v\right)}^{⊤}$.

Consider the case α=0 and notice that for a Riemann problem including a *δ*-shock, the shock speed is extracted from (5a), and from (5b), which determines left and right transport speeds $c_{L}=G(u_{L},\;v)/v$ and $c_{R}=G(u_{R},\;v)/v$. Now, an equation of the transport type $v_{t}+cv_{x}=0$ should be solved at left and right of x=σt, with $c=c_{L}$ and $c_{R}$, respectively. The characteristic lines from (5a) impinge into the shock wave, however, there is no prescribed relationship between the comparisons between *σ* against $c_{L}$ and $c_{R}$, so any of the inequalities (1)-(4) may hold; necessarily the compressibility is preserved with the formation of a shock wave. For *v* we have two transport equations, which can only carry information from the Riemann data; the *δ*-shock is consequence solely of the imbalance of mass at x=σt. Still, this *δ*-shock is surrounded by constant states rather than rarefaction waves.

An overcompressive shock is a restrictive wave in the sense that it is an isolated discontinuity for a Riemann problem connecting left and right states $U_{L}$, $U_{R}$ via this shock; Eq. (1) holds, and there can be neither preceding nor succeeding waves, only constant states on both sides of the discontinuity. Our main result is the construction of the other types of shock waves related to (2)-(4) with a *δ*-shock involved. The new *δ*-shocks may precede or succeed rarefaction waves. Hence, classical Riemann solutions with two wave groups. Typically, there exists an intermediate constant state separating wave groups. The authors in [18] endeavored to produce a set of conservation law models possessing Riemann solutions without such intermediate constant states. Remarkably, the solutions we present here possess a *δ*-shock rather than these intermediate constant states. Other directions are given in [19], where Riemann solutions are reported that possess no intermediate constant states but *δ*-contact discontinuities and, in [5], where interaction of classical waves and *δ*-shocks is given at a positive time.

The rest of this work is organized as follows. In Sec. 1.1, we reconstruct the overcompressive shock wave found by Korchinski. In Sec. 2, we present the new *δ*-shocks of type (2)-(4) with preceding or succeeding central rarefaction fans. Finally, in Sec. 3, we present a Riemann solution possessing two *δ*-shocks. Some concluding remarks are presented in Sec. 4.

### 1.1 The first analysis, back to 1977

Take Korchinski's system [1], and rescale it as in [11]:

(6a) $$u_{t}+{\left(u^{2}\right)}_{x}\;=\;0,\;\;(x,\;t)\in R\times R^{+},$$

(6b) $$v_{t}+{\left(uv\right)}_{x}\;=\;0,\;\;(x,\;t)\in R\times R^{+}.$$

We denote Riemann problems as $\text{RP}(U_{L},\;U_{R})$, comprising a system of conservation laws (as (6a), (6b), (10a), (10b) or (13a), (13b)), and a discontinuous initial condition

(7) $$U(x,\;0)\;=\;\left\{ \begin{array}{cc} U_{L}, & x<0,\\ U_{R}, & x>0. \end{array}\right.$$

From the well-known Rankine-Hugoniot condition, a shock front for *u* with propagation speed $\sigma =u_{L}+u_{R}$ exists when $u_{R}<u_{L}$ holds. In the presence of this shock wave, *v* changes across the front line x−σt=0. The solution profile for v(x,t) can be written as

(8) $$v(x,\;t)\;=\;v_{L}\;+\;(v_{R}-v_{L})H(x-\sigma t)\;+\;k(t)\delta (x-\sigma t),$$

where H is the Heaviside step function and *δ* is the Dirac delta, see for example [5] or for other notations [1], [4], [11]. Here we have taken advantage of the self-similarity property that we are seeking for in a Riemann solution; we know the solution for (6a) and (7) as $u(x,\;t)=u_{L}+(u_{R}-u_{L})H(x-\sigma t)$, [20]. The characteristic speeds satisfy (1) but a simple calculation shows that $v(x,\;t)=v_{L}+(v_{R}-v_{L})H(x-\sigma t)$ does not preserve mass, suggesting the need to add a Dirac delta that compensates for conservation at a “single point”, the front line x−σt=0. However, since the solution is self-similar, it is natural to think that the amplitude of such term will change over time, therefore the k(t) dependency to be determined soon.

In a conservation law, the change of mass in an interval is equal to the net flow of mass at the boundary. For an interval x∈[a,b] with a≪0≪b, the mass balance of v(x,t) in (8) is given by

(9) $$u_{L}v_{L}-u_{R}v_{R}=\frac{d}{dt}\int_{a}^{b}v(x,\;t)\;dx=\frac{d}{dt}\left[\int_{a}^{\sigma t}v_{L}\;dx\;+\;\int_{\sigma t}^{b}v_{R}\;dx\;+\;\int_{a}^{b}k(t)\delta (x-\sigma t)\;dx\right]=\sigma (v_{L}-v_{R})\;+\;k^{\prime }(t).$$

**Figure 1:**
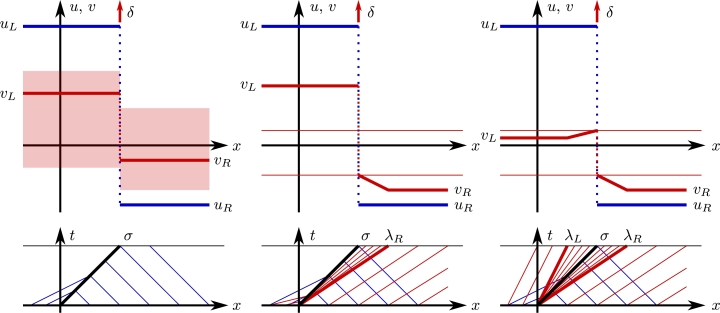
Profiles with *δ*-shocks. We use blue, red and black for curves related to *u*, *v*, and both *u* and *v*. On top, solid lines represent constant states and rarefactions, dotted lines are shock waves at *x* = *σt* (arrows with *δ* are schematic “amplitude” directions of *δ*-shocks); at bottom, we have characteristic speeds on *xt* plane, the horizontal thin line is time *t* = 1 taken as reference for the advance of waves on top panel profiles; *x* = *σt* is in thick dark line, $x=\lambda_{\left\{L,\;R\right\}}t$ are in thick red lines. All Riemann problems have *u*_*L*_ > *u*_*R*_. On left panels is represented the RP for (6a), (6b), shaded regions represent the fact that this configuration exists for any choice of *v*_*L*_, *v*_*R*_. Central and right panels are RP for (10a), (10b), thin horizontal red lines represent the thresholds *λ*_*L*_ = *σ* and *λ*_*R*_ = *σ*; *λ*_*L*_ < *σ* implies a rarefaction before the *δ*-shock as in right panels, similarly *λ*_*R*_ > *σ* implies rarefaction after the *δ*-shock as in central and right panels.

Equating these equalities and integrating over *t* leads to $k(t)=(u_{R}v_{L}-u_{L}v_{R})t$, since the initial condition (7) implies k(0)=0. Thus, this Riemann problem has solution

$$U(x,\;t)\;=\;\left(\begin{array}{c} u_{L}\;+\;(u_{R}-u_{L})H(x-\sigma t)\\ v_{L}\;+\;(v_{R}-v_{L})H(x-\sigma t)\;+\;(u_{R}v_{L}-u_{L}v_{R})t\;\delta (x-\sigma t) \end{array}\right),$$

which is plotted in Fig. 1. The second coordinate state possesses a *δ*-shock with growing amplitude k(t).

Of course, these computations hold in the sense of distributions, see [19], [21], [22]. However, the Riemann solutions in the sections that follow comprise rarefactions that are difficult to handle in these distributions. Even if it is possible to compute the generalized Rankine-Hugoniot conditions given in [6], see also [19], for simplicity we prefer direct computations as in (9).

## 2 A *δ*-shock near a rarefaction wave

In this section we modify system (6a), (6b) in order to produce a richer set of discontinuities around a *δ*-shock. We consider

(10a) $$u_{t}+{\left(u^{2}\right)}_{x}\;=\;0,\;\;(x,\;t)\in R\times R^{+},$$

(10b) $$v_{t}+{\left(uv^{2}\right)}_{x}\;=\;0,\;\;(x,\;t)\in R\times R^{+}.$$

As before, from (10a), a solution for the $\text{RP}(U_{L},\;U_{R})$ has a shock wave with speed $\sigma =u_{L}+u_{R}$ when $u_{R}<u_{L}$; this fact will be assumed from now on.

Now, the nonlinear flux for *v* is $uv^{2}$, so at constant $U_{L,R}$ we have characteristic speeds, $\lambda_{L}=2u_{L}v_{L}$ at the left of the shock front and $\lambda_{R}=2u_{R}v_{R}$ at the right. (The other two characteristic speeds satisfy ${\overset{˜}{\lambda }}_{L}:=2u_{L}>\sigma >2u_{R}=:{\overset{˜}{\lambda }}_{R}$.) In the original model, the flux for *v* is linear around the shock and the *δ*-shock is a consequence of this imposed transport.

New scenarios arise when $\lambda_{L},\;\lambda_{R}>\sigma$ as in Eq. (2), $\sigma >\lambda_{L},\;\lambda_{R}$ as in Eq. (3), or $\lambda_{L}<\sigma <\lambda_{R}$ as in Eq. (4). We study the first and third cases; the second case is similar to the first one. Notice that in the first case, as $\sigma <\lambda_{R}$, the gap in characteristic lines in *xt* plane can be filled with a centered rarefaction fan via the nonlinear flux in (10b). In the third case $\lambda_{L}<\sigma <\lambda_{R}$ hold, thus preceding and subsequent rarefactions appear around the *δ*-shock, see bottom panels in Fig. 1.

### 2.1 The case of *δ*-shock – rarefaction

When the speed inequalities $\sigma <\lambda_{L},\;\lambda_{R}$ hold, at the left of the shock discontinuity, the result must be as in the Korchinski case: $\lambda_{L},\;{\overset{˜}{\lambda }}_{L}>\sigma$. However, at the right of this shock a rarefaction must appear to fill the gap between *σt* and $\lambda_{R}t$ in *xt* plane. For this reason, we take the solution *ansatz*

(11) $$v(x,\;t)=v_{L}\;+\;\left(\frac{x/t}{2u_{R}}-v_{L}\right)H(x-\sigma t)\;+\;\left(v_{R}-\frac{x/t}{2u_{R}}\right)H(x-\lambda_{R}t)+k(t)\delta (x-\sigma t),$$

comprising a “fast” rarefaction that also satisfies (10b). As in (9), the mass balance is computed from (11) as

$$u_{L}v_{L}^{2}-u_{R}v_{R}^{2}=\frac{d}{dt}[\int_{a}^{\sigma t}v_{L}\;dx\;+\;\int_{\sigma t}^{\lambda_{R}t}\frac{x/t}{2u_{R}}\;dx\;+\;\int_{\lambda_{R}t}^{b}v_{R}\;dx+\int_{a}^{b}k(t)\delta (x-\sigma t)\;dx]=\sigma v_{L}\;+\;\frac{\lambda_{R}^{2}-\sigma^{2}}{4u_{R}}\;-\;\lambda_{R}v_{R}\;+\;k^{\prime }(t),$$

which leads to $k(t)=\left[u_{L}v_{L}^{2}-\sigma v_{L}+\sigma^{2}/(4u_{R})\right]t$. An example with $U_{L}={\left(2,\;1\right)}^{⊤}$, $U_{R}={\left(-1,\;-3/4\right)}^{⊤}$ is given in the central panel of Fig. 1.

Borrowing terminology used for Riemann problems for conservation laws (see [14], [23]), we say that this solution is given by a *δ*-shock of type 1-Lax for the first wave group (*i.e.*, the characteristic speeds satisfy (2)), the second wave group is a second family (or fast) rarefaction. This 1-Lax *δ*-shock in the extended sense possesses a Dirac delta with linearly increasing amplitude, as the one in the Korchinski model, see Eq. (9). Moreover, notice the lack of intermediate constant state between wave groups.

### 2.2 The case of rarefaction – *δ*-shock – rarefaction

We consider now the case $\lambda_{L}<\sigma <\lambda_{R}$. The *ansatz* satisfying (10b) is

(12) $$v(x,\;t)=v_{L}\;+\;\left(\frac{x/t}{2u_{L}}-v_{L}\right)H(x-\lambda_{L}t)\;+\;\left(\frac{x/t}{2u_{R}}-\frac{x/t}{2u_{L}}\right)H(x-\sigma t)\;+\;\left(v_{R}-\frac{x/t}{2u_{R}}\right)H(x-\lambda_{R}t)\;+\;k(t)\delta (x-\sigma t),$$

which comprises “slow” and “fast” rarefactions. The mass balance is computed from (12) as

$$u_{L}v_{L}^{2}-u_{R}v_{R}^{2}=\frac{d}{dt}[\int_{a}^{\lambda_{L}t}v_{L}\;dx\;+\;\int_{\lambda_{L}t}^{\sigma t}\frac{x/t}{2u_{L}}\;dx\;+\;\int_{\sigma t}^{\lambda_{R}t}\frac{x/t}{2u_{R}}\;dx\;+\;\int_{\lambda_{R}t}^{b}v_{R}\;dx\;\;+\int_{a}^{b}k(t)\delta (x-\sigma t)\;dx]=\lambda_{L}v_{L}\;+\;\frac{\sigma^{2}-\lambda_{L}^{2}}{4u_{L}}\;+\;\frac{\lambda_{R}^{2}-\sigma^{2}}{4u_{R}}\;-\;\lambda_{R}v_{R}\;+\;k^{\prime }(t),$$

which leads to $k(t)=\sigma^{2}(u_{L}-u_{R})/(4u_{L}u_{R})t$. Notice that stationary shocks, *i.e.* shocks with speed $\sigma =u_{L}+u_{R}=0$, do not produce deltas, since such a delta would have zero amplitude k(t) for all times. An example with σ=1: $U_{L}={\left(2,\;1/8\right)}^{⊤}$, $U_{R}={\left(-1,\;-3/4\right)}^{⊤}$ is given on the right panel of Fig. 1.

This solution is given by a first family (or slow) rarefaction as first wave group, a *δ*-shock of transitional type, see (4), and a second family (or fast) rarefaction as the second wave group. Notice the linear behavior of k(t) and the lack of intermediate constant states between wave groups.

## 3 Example of a wave with two *δ*-shocks

In the previous sections, we have studied wave groups possessing a single *δ*-shock. Our aim now is to construct a new model supporting two of such singular discontinuities. This model possesses the features of models in [4].

**Figure 2:**
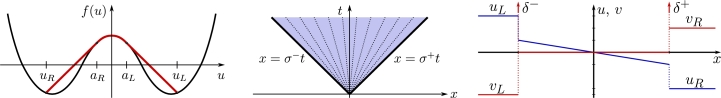
Left: Flux function (14) in black, Oleĭnik convex hull for *u*_*L*_ = 1 = −*u*_*R*_ in red; the envelope is tangent at *a*_*L*_ and *a*_*R*_. Center: Characteristic speeds for the associated RP, solid lines represent shock waves, dotted lines represent centered rarefaction fan. Right: Profile solution for system (13a), (13b); blue is *u*(*x*, *t*) profile, red is *v*(*x*, *t*) profile. Two *δ*-shocks at *σ*^±^*t*, the “amplitudes” are specified; *δ*^±^ denote the pulses *k*_±_(*t*)*δ*(*x* − *σ*^±^*t*).

Let us take a modification of (6a), (6b) with a distinguished conservation for *u* and repeat the conservation law for *v*, see (6b). We write the system

(13a) $$u_{t}+f{\left(u\right)}_{x}\;=\;0,\;(x,\;t)\in R\times R^{+},$$

(13b) $$v_{t}+{\left(uv\right)}_{x}\;=\;0,\;(x,\;t)\in R\times R^{+},$$

where the flux f(u) is a double-well function. For the sake of simplicity, from here and on, we consider

(14) $$f(u)\;=\;\left\{ \begin{array}{cc} {\left(u+2\right)}^{2}-1, & \text{for }\;u<-1,\\ 1-u^{2}, & \text{for }\;u\in [-1,\;1],\\ {\left(u-2\right)}^{2}-1, & \text{for }\;u>1, \end{array}\right.$$

and for the Riemann problem, we consider $u_{L}=-u_{R}=(3+\sqrt{2})/2$. Then, the solution for *u* is

(15) $$u(x,\;t)\;=\;u_{L}\;+\;\left(\frac{x/t}{a_{L}}-u_{L}\right)H(x-\sigma^{-}t)\;+\;\left(u_{R}-\frac{x/t}{a_{L}}\right)H(x-\sigma^{+}t),$$

where from Oleĭnik construction (*cf.* [20] and Fig. 2), we have $\sigma^{+}=-\sigma^{-}=1$ and $a_{R}=-a_{L}=1/2$.

The characteristic speed for *v* is given directly as *u*, thus from (15) we notice that its flux is zero at x=0. The *ansatz* for this system is

(16) $$v(x,\;t)=v_{L}\;+\;(0-v_{L})H(x-\sigma^{-}t)\;+\;(v_{R}-0)H(x-\sigma^{+}t)+k_{-}(t)\delta (x-\sigma^{-}t)\;+\;k_{+}(t)\delta (x-\sigma^{+}t),$$

the solution of which fulfills (13b) and (15). Indeed, the constant regions for $x\notin [\sigma^{-}t,\;\sigma^{+}t]$ satisfy directly $v_{t}={\left(uv\right)}_{x}=0$. For $x\in (\sigma^{-}t,\;\sigma^{+}t)$, we have from (15) and assuming there must be a rarefaction, that it has the form v(x,t)=mx/t for a slope *m* to be specified. Then, by substituting this form into (13b) we obtain

$$v_{t}+{\left(u(x,\;t)\;v\right)}_{x}\;=\;-\frac{mx}{t^{2}}+\frac{1}{a_{L}t}\frac{mx}{t}+\frac{x}{a_{L}t}\frac{m}{t}\;=\;\frac{mx}{t^{2}}\left(-1+\frac{2}{a_{L}}\right)\;=\;0,$$

where the last equality holds only for m=0.

Considering the positive axis, the change of mass of *v* for x≥0 is given from (16) as

$$0-u_{L}v_{L}=\frac{d}{dt}\left[\int_{0}^{\sigma^{+}t}0\;dx\;+\;\int_{\sigma^{+}t}^{b}v_{R}\;dx\;+\;\int_{0}^{b}k_{+}(t)\delta (x-\sigma^{+}t)\;dx\right]=-\sigma^{+}v_{R}\;+\;k_{+}^{\prime }(t).$$

Thus, $k_{+}(t)=(\sigma^{+}v_{R}-u_{R})t$, and $k_{-}(t)\;=\;-(\sigma^{-}v_{L}-u_{L})t$, from an analogous treatment for the change of mass of *v* for x≤0.

In Fig. 2 we plot the solution profile for $\text{RP}(U_{L},\;U_{R})$, where $U_{L}={\left(-(3+\sqrt{2})/2,\;v_{L}\right)}^{⊤}$ and $U_{R}={\left((3+\sqrt{2})/2,\;v_{R}\right)}^{⊤}$, for $v_{L}<u_{R}$ and $v_{R}<u_{L}$; for these settings $k_{+}(t),k_{-}(t)>0$ for all times, the amplitude of both *δ*-shocks is positive.

## 4 Concluding remarks

A crucial feature in constructing the solutions in Sec. 2 is the nonlinear behavior of G(u,v) in *v*, see (5a), (5b). From Eq. (10a), or similar, we can extract the speed *σ*, which determines the existence and localization of *δ*-shocks. The second flux, *i.e.* G(u,v), establishes thresholds by comparing $\lambda_{L}=G_{v}(u_{L},\;v_{L})$ and $\lambda_{R}=G_{v}(u_{R},\;v_{R})$ to *σ*. Notice that $v(x,\;t)\to \lambda_{L}$ ($\lambda_{R}$, respectively) as x→σt− (σt+, resp.), so a “transitional *δ*-shock” has zero amplitude when $\lambda_{L}=\lambda_{R}=\sigma$ hold, but there is a bump at x=0 (typically v(0,t)=0 is larger than $v_{L},\;v_{R}$). In such a situation a *δ*-shock is masked within a bump; small perturbations of the Riemann data will reproduce the linear growing of the delta. In other words, *δ*-shocks can be masked with specific mathematical settings, which stands in contradistinction to their nature from the physical point of view, this reinforces the idea of *δ*-shocks that have not been reported in the literature.

In [24], LeFloch established the existence of solutions for Cauchy problems in a model similar to (13a), (13b) for convex flux f(u). For such fluxes, the Riemann problem may possess a single *δ*-shock. Here we have constructed an elegant solution comprising two *δ*-shocks. In [19], a solution with three *δ*-shocks appears for a 3×3 system of conservation laws. Actually, following the ideas in Sec. 3, we can present a flux f(u) that allows the generation of any number of *δ*-shocks; each contact discontinuity from the Oleĭnik convex hull construction may become a *δ*-shock.

On the other hand, solutions comprising rarefactions and *δ*-shocks were presented in Sec. 2, and we noticed the absence of intermediate constant states in all of them. In the Riemann solutions foreseen in classical theory by Lax and Liu (*cf.* [14], [23]), the existence of intermediate constant states is necessary for the structural stability. Rather, the lack of these states is compensated by *δ*-shocks. In [18], an “organizing center” controls the appearance of transitional waves by eliminating several intermediate constant states, however *δ*-shocks appear in different models, suggesting *δ*-shocks to be a more general phenomenon. In summary, we can build 2×2 Riemann solutions with any number of *δ*-shocks that compose with different elementary waves, such as the transitional shocks used in [25], for long distinguished wave chains. Therefore, we have an eye-catching phenomenon that emerges with potential giving rise to new solutions. These solutions arise in stark contrast of what is known for strictly hyperbolic systems of conservation laws.

## Declarations

### Author contribution statement

P. Castañeda: Conceived and designed the experiments; Performed the experiments; Analyzed and interpreted the data; Contributed reagents, materials, analysis tools or data; Wrote the paper.

### Funding statement

This work was supported by the 10.13039/501100003141CONACYT grant A1-S-26012 and the Asociación Mexicana de Cultura A.C.

### Competing interest statement

The authors declare no conflict of interest.

### Additional information

No additional information is available for this paper.
